# Correction: Nanoporous hybrid CuO/ZnO/carbon papers used as ultrasensitive non-enzymatic electrochemical sensors

**DOI:** 10.1039/d1ra90140k

**Published:** 2021-09-07

**Authors:** Minwei Zhang, Wenrui Zhang, Fei Chen, Chengyi Hou, Arnab Halder, Qijin Chi

**Affiliations:** College of Life Science and Technology, Xinjiang University Urumqi 130046 China zhang78089680@sina.com; Department of Chemistry, Technical University of Denmark DK-2800 Kongens Lyngby Denmark cq@kemi.dtu.dk; The State Key Laboratory for Modification of Chemical Fibers and Polymer Materials, College of Materials Science and Engineering, Donghua University Shanghai 201620 China

## Abstract

Correction for ‘Nanoporous hybrid CuO/ZnO/carbon papers used as ultrasensitive non-enzymatic electrochemical sensors’ by Minwei Zhang *et al.*, *RSC Adv.*, 2019, **9**, 41886–41892. DOI: 10.1039/C9RA08223A.

The author regrets that the funding information was incorrectly shown in the acknowledgements section of the original manuscript. The corrected funding acknowledgement is as shown below.

The author gratefully acknowledges the research facilities provided by the Natural Science Foundation of Xinjiang (No. 2018D01C040).

The Royal Society of Chemistry apologises for these errors and any consequent inconvenience to authors and readers.

## Supplementary Material

